# Optimizing enfortumab vedotin plus pembrolizumab therapy

**DOI:** 10.18632/oncotarget.28741

**Published:** 2025-06-17

**Authors:** Elias Antoine Karam, Yaghi César Céline, Gilles Prince, Fouad Attieh, Hampig Raphael Kourie, Joseph Kattan, Elie Nemer

**Affiliations:** ^1^Departements de Médecine Oncologique, Gustave Roussy F-94805, Villejuif, France; ^2^Department of Hematology-Oncology, Faculty of Medicine, Saint-Joseph University of Beirut, Lebanon; ^3^Department of Urology, Faculty of Medicine, Saint-Joseph University of Beirut, Lebanon; ^*^These authors contributed equally to this work

**Keywords:** advanced urothelial carcinoma (aUC), enfortumab vedotin, pembrolizumab, treatment strategies

## Abstract

Often associated with a poor prognosis, advanced urothelial carcinoma (aUC) has progressed to muscle-invasive or metastatic stages. Traditionally, chemotherapy has been the primary treatment for aUC, though its effectiveness in advanced stages remains limited. Recent developments have introduced promising therapies, notably the combination of enfortumab vedotin with pembrolizumab, which is now recommended as the first-line therapy following the EV-302 trial results. This combination has demonstrated significant improvements in survival rates. This review aims to explore the evolution of treatment strategies for aUC, emphasizing the shift towards immunotherapy and targeted therapies, and discusses the potential for optimized treatment algorithms to improve patient outcomes.

## INTRODUCTION

**Figure 1:**
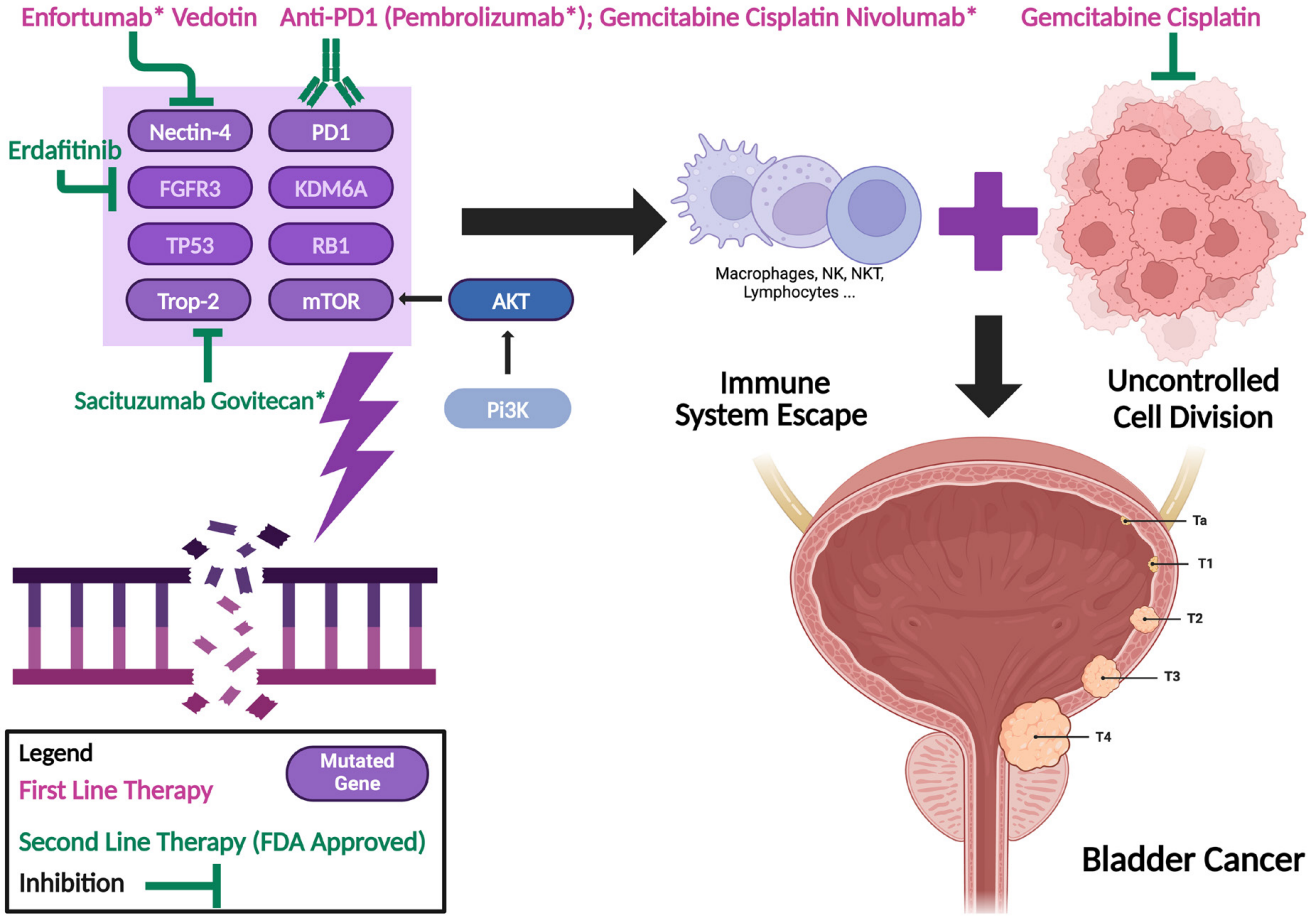
Bladder cancer progression. Genetic alterations, signaling pathways, and targeted therapies; Created in BioRender. Prince, G. (2025) https://BioRender.com/r59h797.

Bladder cancer (BC) is the ninth most common cancer type worldwide, with its incidence approximately four times higher in men than in women [1] with 614298 new cases and 220596 deaths in 2022 [2]. BC arises from the urothelium and accounts for approximately 90% of all urothelial cancers [3]. It progresses through distinct biological processes. Non-muscle invasive bladder cancer (NMIBC), which represents about 75% of BC cases, remains confined to the mucosa and submucosa, often recurring but rarely progressing. In contrast, muscle-invasive bladder cancer (MIBC), comprising about 25% of cases, penetrates the detrusor muscle, increasing the risk of metastasis through lymphatic and vascular invasion. Many key pathways and genetic alterations contribute to its pathogenesis: FGFR3 mutations promote tumor proliferation in NMIBC [4], TP53 and RB1 loss drive genomic instability in MIBC [5]; KDM6A mutations disrupt chromatin remodeling, aiding tumor progression [6]; PI3K-AKT-mTOR activation supports survival and growth [7]; PD-L1 overexpression through PD1 enables immune evasion [8]; Nectin-4 and Trop-2 overexpression enhance tumor adhesion, invasion, and progression, serving as targets for Enfortumab Vedotin and Sacituzumab Govitecan, respectively [9, 10] (Figure 1). Cystoscopy is the gold standard method in detecting BC where suspicious areas can be biopsied. It can be preceded in some cases by urine cytology. MRI, US and CT-Scan can also help in BC staging. Tumor stage (TNM), grade, histological subtype and metastasis are usually the main prognostic factors of urothelial cancer, Metastasis is present in ten to fifteen percent of cases of muscle-invasive BC (MIBC) at time of diagnosis [11]. Moreover, the prognosis for such patients is poor despite recent advancements in treatment regiments. The 5-year survival rate is about 8% at the metastatic stage and about 39% when the tumor extends beyond the bladder and invades adjacent tissue or reaches nearby lymph nodes [12]. The latest trials for advanced-stage urothelial carcinoma (aUC) that introduced new drugs such as antibody-drug conjugates (ADCs) and immune checkpoint inhibitors (ICIs) showed significant improvement in these survival rates. Nevertheless, there is still a major necessity for establishing treatment algorithms that could effectively implement these novel drug combinations. In this article, we will present the major shift in treatment sequences for advanced bladder cancer by starting with the historical standard of care then moving on to the influential impact of enfortumab vedotin plus pembrolizumab and ending with a discussion of other potential treatment strategies.

### Molecular subtypes

Molecular subtypes exhibit distinct clinical outcomes and differ in the expression of cell-cycle genes, cytokeratins, cell adhesion genes, and mutation frequencies. These subtypes transcend pathological classification, with gene signatures consistently expressed across stages and grades, suggesting they are intrinsic tumor properties.

Furthermore, drug susceptibility is more strongly associated with molecular subtypes than pathological classification, highlighting the potential for molecular stratification to guide targeted therapies and personalized treatment approaches.

aUC can be classified into three intrinsic molecular subtypes groups (Table 1): basal, luminal, and neuro-endocrine. The molecular markers identified in these subtypes also exhibit different clinicopathologic characteristics, as well as responses to different therapeutic modalities [13–15].

**Table 1:** Molecular subtypes for aUC

Molecular subtype	Subtype	Expression	Clinical implications	Frequency	Treatment	Genetic mutations
Luminal	**Luminal papillary**	GATA3, uroplakins, CK18, CK20, and, FOXA1, and PPARG), KRT20+	SHH+	Patients <60 years old	35%	FGFR3 inhibitors	FGFR3 mutation, fusion, amplification
	**Luminal non-specified**		EMT markers (TWIST1, ZEB1) miR-200 family, Medium CD274 (PD-L1), CTLA-4 Myofibroblast markers	Patients >80 years old	19%	Anti-PD-L1, PD-1, CTLA-4	Wild type p53
	**Luminal infiltrated**		Immune checkpoint markers (PD-L1, PD-1, and CTLA-4)		6%	Immune checkpoint therapy and radiation therapy	UPKs KRT20 SNX31.
Basal		KRT5/6 and KRT14 CK5/6, CK14, and p63 High CD274 (PD-L1), CTLA4 Immune infiltrates		Advanced or metastatic disease More common in woman	19%	Anti-PD-L1, PD-1, CTLA-4 Cisplatin-based chemotherapy EGFR targeted therapy	
Neuro-endocrine				Most aggressive	5%	Etoposide Cisplatin-based chemotherapy	SOX2 DLX6 MSI1 PLEKHG4B E2F3/SOX4 amplification High cell cycle

### Histological subtypes

The histological subtypes (Table 2) of urothelial carcinoma are classified as follows: infiltrating urothelial carcinoma with divergent differentiation; nested, microcystic; micropapillary; lymphoepithelioma-like; plasmacytoid/signet ring cell/diffuse; sarcomatoid; giant cell; poorly differentiated; lipid-rich; and clear cell. Each subtype carries unique prognostic and therapeutic implications, making accurate classification essential for effective management and treatment [16].

**Table 2:** Histological subtypes for aUC

Histological subtype	Corresponding molecular subtype	Genetic alterations	Marker expression	Clinical implications	Prognosis
Plasmacytoid Urothelial Carcinoma	Luminal and Basal	TP53, RB1, KMT2D, ARID1A mutations, CDH1 loss-of-function mutations, CDH1 promoter hypermethylation, loss of E-cadherin, abnormal expression of p120		Advanced stage at presentation High relapse rates Peritoneal carcinomatosis	Poor survival, High cancer-specific mortality High risk of recurrence and metastasis Local recurrences
Micropapillary Urothelial Carcinoma	Luminal	ERBB2 Amplifications, PPARG enrichment and suppression of p63 target genes	Downregulation of miR-296 and activation of chromatin-remodeling complex RUVBL1	High-grade tumor cells Intra-tumoral heterogeneity Vascular invasion and nodal metastasis	Poor survival Intratumoral heterogeneity complicates prognosis
Small-Cell/ Neuroendocrine Carcinoma	Luminal and Basal	TP53, RB1 mutations; TERT promoter mutations, chromatin-remodeling gene mutations (CREBBP, EP300, ARID1A, KMT2D, APOBEC) APOBEC mutation signature, high level of chromosomal instability and genomic doubling	Neuroendocrine markers CD56, synaptophysin, chromogranin, and INSM1, NEUROD1, ASCL1, POU2F3, YAP1, and DLL3	De novo neuroendocrine differentiation Association with paraneoplastic syndromes Aggressive course	Poor response to treatment Frequent disseminated metastasis
Sarcomatoid Urothelial Carcinoma	Basal	TP53, RB1, PIK3CA mutations; dysregulation of epithelial–mesenchymal transition pathway	High molecular keratins CK 34ßE12 or CK5/6; and or (3) GATA3 expression in the sarcomatous areas.	Aggressive Bi-phasal	Extremely poor prognosis due to high invasiveness and aggressive behavior
Squamous cell carcinoma	Basal	TERT promoter mutations	Basal and stem-like markers (CD44, CK5, CK6, and CK14), epidermal growth factor receptor (EGFR) and desmocollins (DSC1-3) and desmogleins (DSG1–4), TGM1 (transglutaminase 1), and PI3 (elafin)		Unfavorable prognosis High-grade urothelial carcinoma Poor response to chemotherapy and radiation
Nested Urothelial Carcinoma		TERT promoter mutations, *TP53*, *JAK3*, *CTNNB1, FGFR3*	FOXA1, GATA3, and CK20, PAX8 expression	Aggressive clinical course	Variable prognosis Aggressive behavior High frequency of metastasis
Urothelial carcinoma with glandular differentiation		*TERT* promoter, chromatin-modifying genes, and DNA damage response (DDR) genes.	MUC5AC and CDX2	High stage at presentation	
Adenocarcinoma		TP53, KRAS, SMAD4 (similar to colorectal adenocarcinoma), EGFR and ERBB2 amplification	CK20 and CDX2	Pure glandular morphology Resembles colorectal adenocarcinomas Intestinal metaplasia, bladder exstrophy, chronic irritation, and obstruction due to nonfunctioning bladder or endemic schistosomiasis.	Poor prognosis High-grade urothelial carcinoma Poor response to chemotherapy

This table summarizes the most common histological subtypes of urothelial carcinoma, detailing their specific genetic alterations, clinical implications, and prognosis. It highlights the distinct features of each subtype, emphasizing the importance of accurate classification in guiding treatment decisions and predicting patient outcomes [17–19].

### Predictive biomarkers

Recent biomarkers have been identified to predict treatment response and prognosis in aUC. They play a key role in personalized treatment, emphasizing the need for further research. Table 3 summarizes the most significant biomarkers, while others, including PARP, HER2, HER1, ERCC1, and ERCC2, show potential for guiding future therapies or serving as prognostic indicators in aUC [20, 21].

**Table 3:** Predictive and prognostic biomarkers in aUC

Biomarker	Mechanism of action	Role	Drug	Drug mechanism of action
FGFR	Promote angiogenesis and the regeneration of tissue in cellular proliferation, differentiation and steroid synthesis.	Predictive of response to FGFR inhibitors	Erdafitinib	Pan-FGFR tyrosine kinase inhibitor [22]
TMB		Predictive of response to immunotherapy (PD-1 inhibition) Prognostic factor	Pembrolizumab	Block immune-suppressing ligands (PD-L1 and PD-L2), from interacting with PD-1 to help restore T-cell response and immune response [23]
Nectin-4	Cell-cell adhesion, proliferation, angiogenesis, epithelial to mesenchymal transition, metastasis, DNA repair, tumor relapse	Potential predictive biomarker for response to ADC	Enfortumab Vedotin	Binds to cells expressing Nectin-4, leading to internalization of the ADC-Nectin-4 complex. The MMAE is then released through proteolytic cleavage, where it induces cell cycle arrest and apoptotic cell death [24]
Trop-2	Cell proliferation, survival and invasion	Potential predictive factor for Sacituzumab govitecan response Prognostic factor	Sacituzumab govitecan	The delivery of SN-38 to the tumor cell results in inhibition of topoisomerase I and the accumulation of lethal DNA double strand break [25]

## THE STANDARD OF CARE BEFORE EV-302

### First line therapy

For the past decades, platinum-based chemotherapy has served as the standard frontline therapy for patients with aUC, with cisplatin preferred over carboplatin in the first-line setting. In fact, treatment algorithm selection depends on the patient’s tolerance to platinum-based cytotoxic drugs, more specifically to cisplatin. Numerous first-line combinations for cisplatin-eligible patients have been investigated in historical clinical trials during the last thirty years. The MVAC regimen, comprising methotrexate, vinblastine, doxorubicin, and cisplatin, administered every 28 days for six cycles, demonstrated significant improvements in objective response rate (ORR), PFS, and OS compared to single-agent cisplatin in aUC [26]. MVAC therapy is associated with significant toxicity, including myelosuppression, neutropenic fever, sepsis, mucositis, and nausea and vomiting. These considerable adverse effects led to establishing different combinations, such as dose-dense MVAC and gemcitabine plus cisplatin, to circumvent these marked side effects.

While dose-dense MVAC did not significantly improve OS compared to classic MVAC, it demonstrated benefits in terms of PFS and toxicity reduction. Compared to classic MVAC, dose-dense MVAC showed a more favorable toxicity profile, with lower rates of grade ≥3 leukopenia, mucositis, and neutropenic fever [27–29] In a phase III trial comparing gemcitabine plus cisplatin (GC) with classic MVAC [29, 30], GC regimen has shown comparable efficacy and reduced toxicity compared to the MVAC regimen, making it a promising alternative, GC demonstrated similar ORR and OS outcomes. GC was associated with less grade ≥3 toxicity than MVAC, including lower rates of neutropenia, neutropenic sepsis, and mucositis.

These multiple first-line cisplatin-based combinations were recommended for aUC as a standard of care for multiple years although GC remained the most commonly used regimen. However, nearly half of patients are ineligible for cisplatin use due to frequent medical comorbidities and/or reduced renal function [31]. Thus, the addition of carboplatin to gemcitabine was recommended as the first-line therapy for these cases. It is particularly suitable for those who cannot tolerate more complex carboplatin-based combination regimens like MCAVI (methotrexate, carboplatin, and vinblastine).

The evidence for this indication comes from the randomized phase II/III EORTC 30986 trial [32] that involved chemotherapy-naïve patients with advanced or metastatic UC and impaired kidney function or poor performance status. The gemcitabine plus carboplatin combination was compared with MCAVI. The study found that treatment with carboplatin plus gemcitabine resulted in similar OS and PFS compared to MCAVI. Although the ORR was slightly higher with gemcitabine plus carboplatin, the difference was not statistically significant. Gemcitabine plus carboplatin was associated with lower rates of grade 3 to 4 toxicity compared to MCAVI, particularly in terms of neutropenia and febrile neutropenia. However, it was linked to a higher incidence of serious thrombocytopenia.

For patients unfit for both cisplatin and carboplatin, immunotherapy for PD-L1-positive cases such as pembrolizumab is recommended [33]. In the phase II KEYNOTE-052 [34] study, pembrolizumab demonstrated significant efficacy as initial therapy in patients with aUC who were ineligible for a cisplatin-based regimen. The ORR for the entire cohort was 29%, with complete and partial response rates of 9% and 20%, respectively. Importantly, the median duration of response was 33 months, indicating durable responses. Response rates remained consistent across various subgroups, with higher ORR observed in patients with a combined positive score (CPS) >10 compared to CPS ≤10. The median OS was 11 months, with a four-year OS rate of 19%. Finally, combination therapy involving nivolumab alongside gemcitabine–cisplatin demonstrated superior outcomes compared to gemcitabine–cisplatin alone in the CheckMate 901 trial [35]. The positive overall survival findings of this study led to a recent FDA approval of this combination as a first-line treatment for unresectable or metastatic urothelial carcinoma [36].

### Maintenance therapy

Although platinum-based cytotoxicity was cemented as the optimal first-line approach for increasing patient survival, the poor durability of response highlighted the need for first-line maintenance therapy in cases without disease progression. As a result of the JAVELIN Bladder 100 phase III trial, avelumab first-line maintenance therapy has been integrated into international guidelines as a standard of care for patients with aUC who do not experience progression following first-line platinum-based chemotherapy. In the avelumab group, the median overall survival (OS) was 21.4 months, compared to 14.3 months in the control group (BSC). Additionally, the median progression-free survival (PFS) was 3.7 months in the avelumab group and 2.0 months in the control group [37, 38]. While there have been notable improvements observed with avelumab maintenance treatment, it is evident that survival rates remain relatively low.

### Second-line therapy

Several innovative treatments such as FGFR inhibitors, ADCs, and ICIs [39–41] were implemented as second-line therapies for aUC patients with disease progression after receiving standard-of-care first-line drugs.

Erdafitinib, a FGFR inhibitor, demonstrates promising efficacy as a second-line drug in patients with advanced or metastatic UC harboring a FGFR3 genetic alteration. Initial efficacy, supported by early phase II clinical trials, included an ORR of 40% and a median OS of 11 months [39]. Furthermore, in the phase III THOR trial [22], erdafitinib showed significant improvements in OS and PFS compared to chemotherapy, with benefits observed across all clinically relevant subgroups. Erdafitinib is generally well tolerated in long-term treatment, maintaining patient quality of life. Mutations in FGFR3, AKT1, and TP53, detected in cfDNA may contribute to acquired resistance to erdafitinib [42].

Enfortumab vedotin, an ADC targeting Nectin-4, demonstrated significant improvements in OS and PFS compared to chemotherapy in the randomized phase III EV-301 trial. In this study involving 608 patients with locally advanced unresectable or metastatic UC previously treated with platinum-based chemotherapy and PD-1/PD-L1 inhibitor, enfortumab vedotin exhibited superior OS (median 13 vs. 9 months) and PFS (median 6 vs. 4 months) compared to chemotherapy [43, 44]. Additionally, overall response rates were higher with enfortumab vedotin than with chemotherapy (41% vs. 19%). Sacituzumab govitecan, an antibody-drug conjugate targeting Trop-2, demonstrated promising efficacy in a phase II trial (TROPHY-U-01) involving 113 patients with advanced UC previously treated with platinum-based chemotherapy or immunotherapy. The study reported objective and complete response rates of 27% and 5%, respectively, with median OS and median PFS of 5 and 11 months, respectively [45].

Pembrolizumab was also a potential therapeutic alternative for relapsed aUC as shown in the KEYNOTE-045 trial. This ICI improved median OS compared to paclitaxel, docetal, or vinflunine (10.3 vs. 7.4 months, HR 0.73, 95% CI 0.59–0.91) in recurrent aUC cases. The durability of response was also demonstrated in a three-year follow-up update [46, 47].

## THE CHANGING TREATMENT LANDSCAPE AFTER EV-302 AND CHECKMATE 901

After years of employing platinum-based established protocols, the EV-302 trial served as a pivotal milestone that introduced ADCs into the first-line armamentarium against aUC. Enfortumab vedotin plus pembrolizumab is now approved as a first-line treatment regardless of cisplatin-eligibility [48]. The CheckMate-901 trial also prompted a significant shift in the treatment algorithm by proving an increased survival with the use of nivolumab with the standard gemcitabine-cisplatin combination in the first-line [35, 49], this could be useful for patients who cannot receive enfortumab vedotin plus pembrolizumab. In this section, we will discuss the intricacies of these clinical trials and the implications behind the major change in the treatment sequences.

### The favored regimen of enfortumab vedotin plus pembrolizumab

For patients diagnosed with aUC, it is recommended starting treatment with enfortumab vedotin in conjunction with pembrolizumab over platinum-based [35, 50].

Enfortumab vedotin is a fully human ADC consisting of a human IgG1 antibody targeting Nectin-4, linked to monomethyl auristatin E (MMAE), a microtubule-disrupting agent. Its anticancer effect occurs when the ADC binds to cells expressing Nectin-4, leading to internalization of the ADC-Nectin-4 complex. The MMAE is then released through proteolytic cleavage, where it induces cell cycle arrest and apoptotic cell death [24]. Nectin 4 is expressed in more than 90% of urothelial carcinoma [51]. The luminal subtype of urothelial carcinoma is the one who expresses Nectin 4 the most. Resistance to this treatment includes down regulation or knockdown of NECTIN4 [52].

The most common adverse events associated with EV therapy include peripheral sensory neuropathy, pruritus, fatigue, reduced appetite, diarrhea, dysgeusia, and nausea [53].

Pembrolizumab is a programmed death 1 (PD-1) inhibitor that binds to the PD-1 receptor, blocking immune-suppressing ligands (PD-L1 and PD-L2), from interacting with PD-1 to help restore T-cell response and immune response.

PD-L1 and PD-L2 bind to PD-1, a receptor expressed on activated and exhausted T cells, as well as on antigen-presenting cells like macrophages, dendritic cells, and B cells. When PD-L1 interacts with PD-1, it triggers phosphorylation of the immunoreceptor tyrosine-based switch motif within the intracellular domain of PD-1, leading to the recruitment of SHP-1 and SHP-2 phosphatases. These phosphatases modulate kinases associated with the T-cell antigen receptor, thereby reducing cytokine production, T-cell activation, and target cell lysis [23].

Various biomarkers defining the tumor microenvironment may aid in predicting the response to pembrolizumab monotherapy in aUC. For example, TMB and Tcell_inf_GEP show a correlation with better outcomes [54].

The most frequent long term adverse events to a treatment with Pembrolizumab include Fatigue, Pruritus, Rash, Decreased appetite, Hypothyroidism, Diarrhea and Nausea [55].

The phase III EV-302 trial [56] involving 886 patients with previously untreated, locally advanced or metastatic UC demonstrated superior OS at a median follow-up duration of 17.2 months (31.5 months vs. 16.1 months, HR 0.47, 95% CI 0.38–0.58), PFS (12.5 months vs. 6.3 months, HR 0.45, 95% CI 0.38–0.54), and response rates with enfortumab vedotin plus pembrolizumab compared to chemotherapy [56, 57].

This efficacy extended to cisplatin-eligible and cisplatin-ineligible patients, with significant improvements in OS and PFS across various clinically relevant subgroups. Grade ≥3 toxicities were lower with enfortumab vedotin plus pembrolizumab compared to platinum-based chemotherapy, with manageable profiles. However, it’s important to note that while these toxicities were reported less, the resulting profiles from these complications differ significantly. Therefore, long-term studies and patient-reported outcomes are crucial in determining the long-term management and prognosis. The findings of this trial led to an FDA approval in December 2023 for first-line aUC indication of enfortumab vedotin plus pembrolizumab irrespective of cisplatin-eligibility [48].

In the case of disease progression after enfortumab vedotin plus pembrolizumab, an optimal approach to second-line therapy remains uncertain. For patients eligible for cisplatin, it is advised to opt for cisplatin-based chemotherapy, while using the treatment options previously discussed, although data on this context are limited. If patients are ineligible for cisplatin, gemcitabine plus carboplatin represents a suitable alternative, although data on this context are limited. Moreover, further research is needed to determine the efficacy of avelumab maintenance treatment following platinum-based therapy after pembrolizumab plus enfortumab vedotin. The ESMO Clinical Practice Guideline 2024 interim update states that second-line platinum-based combinations should be administered without avelumab in FGFR negative cases. For example: Sacituzumab Govitecan can be administered or Enfortumab Vedotin if not already administered. Whereas if the patient has FGFR mutations, Erdafitinib can be administered. In addition, single ICI rechallenge is also not advised without upcoming evidence [58]. For individuals ineligible for platinum-based therapy, the investigation of FGFR2/3 mutations remains crucial in the pursuit of suitable treatment options such as erdafitinib. Ultimately, defining second-line therapy after this novel combination remains challenging; it is still unclear whether this sequence could provide better survival benefits in comparison to the previous GC-avelumab followed by enfortumab vedotin sequence.

### Treatment sequences for platinum-based regimens

The recommendation for initial therapy in aUC favors enfortumab vedotin plus pembrolizumab due to its superior efficacy and manageable toxicity profile observed in clinical trials. However, patients with the following conditions are ineligible for treatment with pembrolizumab plus enfortumab vedotin [56]: uncontrolled diabetes mellitus, severe dermatologic conditions, grade ≥2 neuropathy, creatinine clearance ≤30 mL/minute or failure of immunotherapy in the adjuvant setting. Moreover, the economic considerations [57] surrounding the utilization of enfortumab vedotin and pembrolizumab cannot be overlooked. A recent analysis [59] revealed that the estimated annual cost of treatment with these agents was 3.8 times higher compared to platinum-based chemotherapy followed by avelumab maintenance ($455,630 vs. $120,253). Considering that certain patients may not be suitable candidates, decline treatment, or lack access to the initial therapy, alternative options must be explored.

Patients who cannot receive enfortumab vedotin plus pembrolizumab will benefit from the previous platinum-based standard of care with the addition of a novel combination evaluated in the CheckMate 901 trial. After the negative findings of Keynote 362 and IMvigor 130, this study was the first to demonstrate overall survival benefits in the case of a first-line GC-ICI combination. In the CheckMate 901 phase III trial, 608 patients were randomized (1:1) into receiving either gemcitabine-cisplatin plus nivolumab every 3 weeks for a maximum of 6 cycles followed by up to 24 months of nivolumab monotherapy (every 4 weeks) or gemcitabine-cisplatin alone for a maximum of 6 cycles. After a median follow-up duration of 33.6 months, adding nivolumab to gemcitabine-cisplatin (GCN) significantly improved survival compared to GC alone. Notably, OS was prolonged with GCN therapy, with a median OS of 21.7 months compared to 18.9 months with GC alone. PFS was also extended with nivolumab-combination therapy, with a median PFS of 7.9 months versus 7.6 months. It should be noted that these positive survival results were present irrespective of PD-L1 expression of the tumor. This trial demonstrated that the GCN combination was also responsible for an early and durable response as seen in the ORR rates (58% for the GCN arm vs. 43% for the GC arm). The GCN group showed a higher complete response (CR) rate than the GC group (22% vs. 12%), this response was also more durable (median duration of CR: 37.1 months vs. 13.2 months). However, it’s important to note that grade ≥3 toxicity was more prevalent in the GCN group, primarily involving neutropenia and thrombocytopenia [35, 50]. Patients who received GCN as a first-line treatment for aUC can benefit from maintenance nivolumab therapy [29].

In the case of lack of access to CGN or ineligibility, it is recommended that patients receive the previously established standard of care that consisted of gemcitabine with the addition of either cisplatin or carboplatin (based on cisplatin eligibility). Avelumab maintenance therapy is advised in the absence of disease progression.

If the patient experiences disease recurrence, several second-line options can be proposed. Pembrolizumab has shown favorable results in recurrent aUC after platinum-based chemotherapy in the Keynote-045 trial and could be recommended as a second-line treatment in ICI-naïve cases. It is preferred that patients who have received ICI (such as nivolumab) with platinum-based regimens in the first line and experience disease recurrence benefit from erdafitinib depending on FGFR DNA fusions and mutations or enfortumab vedotin [58]. Sacituzumab govitecan has been also proven to be effective in cases with disease progression after receiving platinum chemotherapy with PD-1/PD-L1 inhibitors as shown in the TROPHY-U-01 trial [45].

### Recommendations for platinum-ineligible patients

Immune checkpoint inhibitor monotherapy can be recommended in the first-line if patients are unfit or lack access to enfortumab vedotin plus pembrolizumab or platinum-based regimens. Pembrolizumab monotherapy in this case is indicated based on the KEYNOTE-052 phase II study which has proven positive response rates as previously mentioned in the first section [34]. After experiencing disease relapse post-ICI monotherapy, patients can benefit from enfortumab vedotin based on the findings of the EV-201 phase II trial. In this study comprising 91 such patients, enfortumab vedotin yielded an ORR of 52%, with complete and partial response rates of 20% and 31% respectively [60].

**Figure 2:**
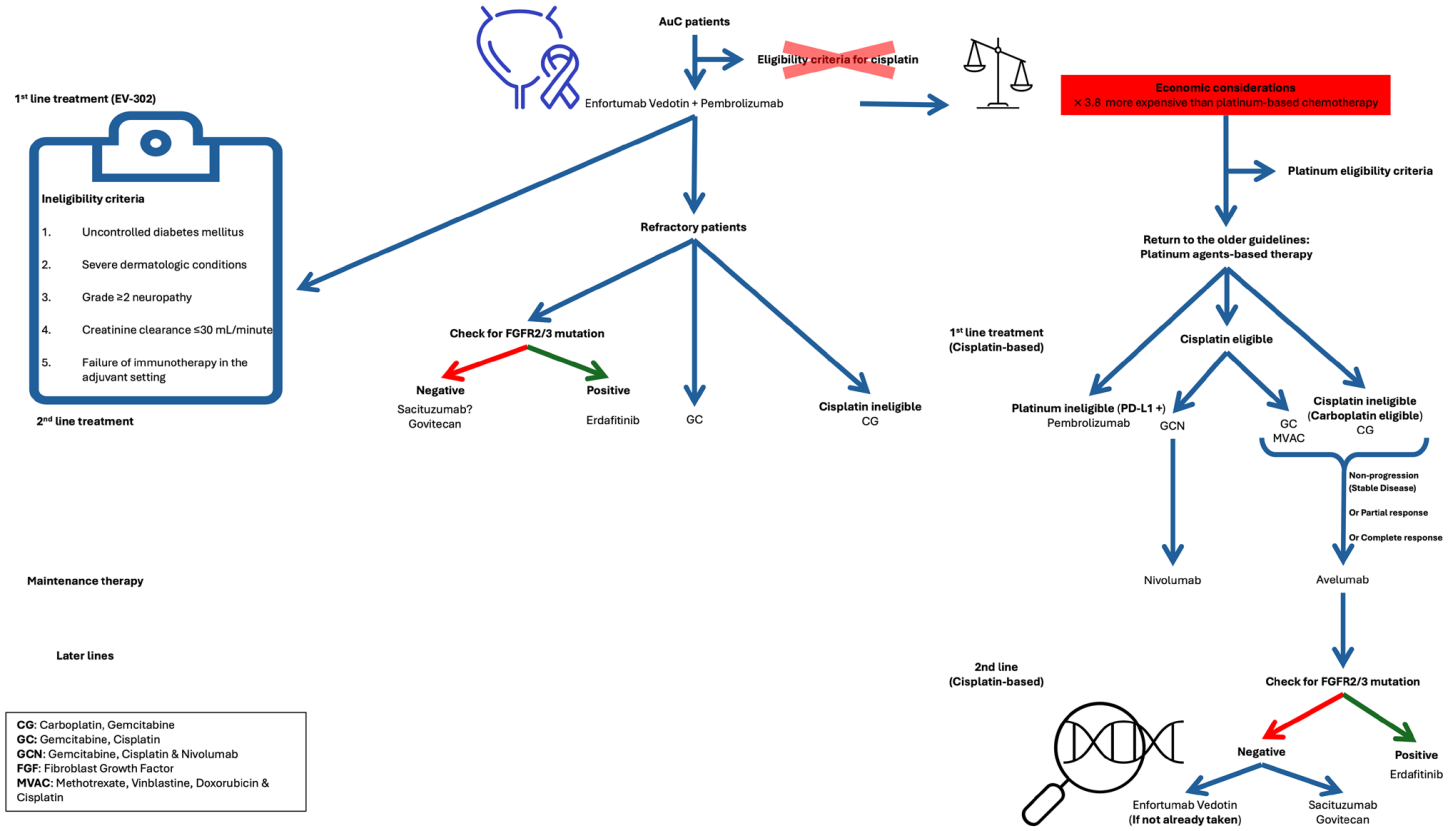
New sequence of treatment based on Phase III trial (EV-302). Bladder cancer Icon made by cube29 from https://www.flaticon.com/.

Further research is needed to determine the optimal second-line treatment following the first-line combination of Pembrolizumab and Enfortumab Vedotin. In addition to efficacy, factors such as cost differences and toxicity profiles must be considered when selecting subsequent therapies. Figure 2 presents a proposed algorithm outlining potential treatment sequences for advanced urothelial carcinoma (aUC).

## UNCHARTED WATERS: NAVIGATING OBSTACLES AFTER EV-302

With the introduction of novel combinations into the treatment arsenal against advanced-stage urothelial carcinoma, several dilemmas arise, and questions remain unanswered. For instance, is there a specific population more likely to respond to pembrolizumab + enfortumab vedotin as a first-line treatment? Subgroup analyses are necessary to correctly stratify patients and ensure an effective personalized treatment plan. Moreover, there is still uncertainty around the efficacy of these new combinations in subgroups that have already received adjuvant immunotherapy.

Predictive and prognostic biomarkers are also lacking in the aUC sphere, with an unmet need for markers such as ctDNA that could help modify the administered medication in terms of escalation or cessation. Liquid biopsy could in fact aid in monitoring disease progression in a non-invasive manner which could lead to a possible reduction of adverse effects. ctDNA is secreted into the bloodstream by apoptotic tumor cells. It is used in urothelial carcinoma for MRD assessment and follow-up after surgical treatment and chemotherapy [61, 62]. Further research needs to be done concerning the use of ctDNA monitoring in patients receiving the combination of pembrolizumab and enfortumab vedotin.

Ultimately, the increasing cost of such drugs and the potential resistance mechanisms that have not yet been elucidated are challenges that could arise with the new adoption of these combinations. New clinical trials are underway to validate the effectiveness of this combination, including EV-304, a phase 3 study evaluating the regimen of Pembrolizumab + Enfortumab Vedotin against the standard Neoadjuvant Gemcitabine and Cisplatin in Cisplatin-eligible participants with muscle-invasive bladder cancer. The primary endpoint is the Event-Free Survival [63]. Another ongoing clinical trial, EV-303, is a phase 3 study with three arms: Pembrolizumab + Surgery, Surgery alone, and Enfortumab Vedotin + Pembrolizumab + Surgery. It involves participants with muscle-invasive bladder cancer (MIBC) who are either ineligible for cisplatin or have declined it. The primary endpoint is the Event-Free Survival [64]. Other trials with published results are represented in Table 4.

**Table 4:** Pivotal clinical trials in patients with aUC

Trial name	Agents	Type	Setting	Year	Trial	ORR/mPFS/mOS	Toxicities
EV-302; NCT04223856	Enfortumab, Vedotin and Pembrolizumab	ADC	First line	2024	Phase III [56]	−/12.5 mo/31.5 mo”	Peripheral neuropathy, ocular, pulmonary, and cutaneous toxicities, hyperglycemia, and immune-related adverse events
−	MVAC	Cytotoxic and anti-angiogenic	First line, cisplatin-fit	1992	[26]	39%/10 mo/12.5 mo	Myelosuppression, neutropenic fever, sepsis, mucositis, and nausea and vomiting
Protocol No. 30924	Gemcitabine and cisplatin	Cytotoxic	First line, cisplatin-fit	2000 and 2005	Phase III [27]	49%/7.4 mo/13.8 mo and −/7.7 mo/14 mo	Lower rates of neutropenia, neutropenic sepsis, and mucositis
NCT03036098 ONO-4538-X41	Cisplatin, Gemcitabine and Nivolumab	Cytotoxic and ADC	First line, cisplatin-fit	2023	Phase III [35, 50]	57.6%/7.9 mo/21.7 mo	Neutropenia and thrombocytopenia
EORTC study 30986	Gemcitabine and Carboplatin	Cytotoxic	First line, cisplatin-unfit	2011	Phase II/III [32]	42.1%/5.8 mo/9.3 mo	lower rates of neutropenia and febrile neutropenia, but higher incidence of serious thrombocytopenia
KEYNOTE-052 NCT02335424	Pembrolizumab	Anti-PD-L1	First line, platinum-unfit	2017	Phase II [55]	29%/2 mo/11 mo	Incidence of severe or life-threatening toxicities is low
JAVELIN Bladder 100 trial NCT02603432	Avelumab	Anti-PD-L1	Maintenance therapy	2023	Phase III [38, 65]	−/5.5 mo/23.8 mo	Urinary tract infection, diarrhea, and arthralgias
BLC2001 study; NCT02365597 NCT03390504	Erdafitinib	FGFR inhibitor	Later lines, after platinum therapy and immunotherapy, FGFR2/3 positive patient	2022 and 2023	Phase II [40] and Phase II [22]	40%/−/11 mo and 45.6%/5.6 mo/12.1 mo	Palmar-plantar erythrodysesthesia, stomatitis, onycholysis, hyperphosphatemia, and diarrhea
EV-301	Enfortumab vedotin	FGFR inhibitor and Cytotoxic	Later lines, after platinum therapy and immunotherapy, FGFR2/3 negative patient	2022	Phase III [44, 53]	41%/6 mo/13 mo	Rash, peripheral neuropathy, and hyperglycemia, ocular toxicities, pneumonitis, and severe cutaneous adverse reactions
TROPHY-U-01; NCT03547973	Sacituzumab and govitecan	Antibody-drug conjugate targeting Trop-2	Later lines, after platinum therapy and immunotherapy, FGFR2/3 negative patient	2021	Phase II [45]	27%/11 mo/5 mo	Neutropenia, leukopenia, anemia, diarrhea, and febrile neutropenia
EV201; NCT03219333	Enfortumab and vedotin	FGFR inhibitor and Cytotoix	Later lines, cisplatin-unfit therapy after immunotherapy, FGFR2/3 negative patient, refractory aUC	2021	Phase II [60]	52%/5.8 mo/14.7 mo	−

Abbreviations: ADC: antibody-drug conjugate; mo: months; MVAC: Methotrexate, Vinblastine, Doxorubicin and Cisplatin; FGF: fibroblast growth factor; ORR: objective response rate; mPFS: median progression-free survival; mOS: median overall survival.

In conclusion, the management of aUC has seen notable advancements, offering patients a variety of promising treatment options aimed at enhancing outcomes. Recent research has highlighted the effectiveness of pembrolizumab and enfortumab vedotin, solidifying their position as compelling choices for first-line therapy. Nevertheless, there is a pressing need for further investigation and clinical trials to better understand the implications of implementing these treatments as initial interventions. Continued efforts in research and collaboration are crucial to improve treatment strategies for individuals with aUC.

## Abbreviations

ADCs: Antibody-drug conjugates
aUC: Advanced-stage urothelial carcinoma
BC: Bladder cancer
CPS: Combined positive score
CR: Complete Response
FGFR: Fibroblast Growth Factors
GC: Gemcitabine-cisplatin
GCN: Gemcitabine-cisplatin-nivolumab
ICIs: Immune checkpoint inhibitors
MCAVI: Methotrexate, carboplatin, and vinblastine
MIBC: Muscle-invasive bladder cancer
NMIBC: Non-muscle invasive bladder cancer
MVAC: Methotrexate, vinblastine, doxorubicin, and cisplatin
ORR: Objective response rate
OS: Overall survival
PFS: Progression-free survival
UC: Urothelial carcinoma

## References

[1] Lobo N , Afferi L , Moschini M , Mostafid H , Porten S , Psutka SP , Gupta S , Smith AB , Williams SB , Lotan Y . Epidemiology, Screening, and Prevention of Bladder Cancer. Eur Urol Oncol. 2022; 5:628–39. 10.1016/j.euo.2022.10.003. 36333236

[2] Cancer Today. https://gco.iarc.who.int/today/.

[3] Key Statistics for Bladder Cancer. https://www.cancer.org/cancer/types/bladder-cancer/about/key-statistics.html.

[4] Neuzillet Y , van Rhijn BW , Prigoda NL , Bapat B , Liu L , Bostrom PJ , Fleshner NE , Gallie BL , Zlotta AR , Jewett MA , van der Kwast TH . FGFR3 mutations, but not FGFR3 expression and FGFR3 copy-number variations, are associated with favourable non-muscle invasive bladder cancer. Virchows Arch. 2014; 465:207–13. 10.1007/s00428-014-1596-4. 24880661

[5] Manzano RG , Catalan-Latorre A , Brugarolas A . RB1 and TP53 co-mutations correlate strongly with genomic biomarkers of response to immunity checkpoint inhibitors in urothelial bladder cancer. BMC Cancer. 2021; 21:432. 10.1186/s12885-021-08078-y. 33879103 PMC8056512

[6] Matar M , Prince G , Hamati I , Baalbaky M , Fares J , Aoude M , Matar C , Kourie HR . Implication of *KDM6A* in bladder cancer. Pharmacogenomics. 2023; 24:509–22. 10.2217/pgs-2023-0027. 37458596

[7] Sathe A , Nawroth R . Targeting the PI3K/AKT/mTOR Pathway in Bladder Cancer. Methods Mol Biol. 2018; 1655:335–50. 10.1007/978-1-4939-7234-0_23. 28889395

[8] Rouanne M , Radulescu C , Adam J , Allory Y . PD-L1 testing in urothelial bladder cancer: essentials of clinical practice. World J Urol. 2021; 39:1345–55. 10.1007/s00345-020-03498-0. 33141317

[9] Aoki M , Naiki T , Naiki-Ito A , Morikawa T , Matsuyama N , Torii K , Kato T , Maruyama T , Inaguma S , Yasui T . Successful treatment with enfortumab-vedotin of metastatic signet ring cell cancer expressing nectin-4 and originating from the bladder. IJU Case Rep. 2023; 7:110–14. 10.1002/iju5.12678. 38440703 PMC10909144

[10] Abbas M , Heitplatz B , Bernemann C , Boegemann M , Trautmann M , Schrader AJ , Wardelmann E , Schlack K . Immunohistochemical expression of TROP-2 (TACSTD2) on the urothelial carcinoma of the urinary bladder and other types of cancer. Oncol Lett. 2023; 26:527. 10.3892/ol.2023.14114. 38020299 PMC10644361

[11] Rosenberg JE , Carroll PR , Small EJ . Update on chemotherapy for advanced bladder cancer. J Urol. 2005; 174:14–20. 10.1097/01.ju.0000162039.38023.5f. 15947569

[12] Cancer. Net. 2012. Bladder Cancer - Statistics. https://www.cancer.net/cancer-types/bladder-cancer/statistics.

[13] Sun M , Trinh QD . Diagnosis and staging of bladder cancer. Hematol Oncol Clin North Am. 2015; 29:205–18. 10.1016/j.hoc.2014.10.013. 25836929

[14] Guo CC , Czerniak B . Molecular Taxonomy and Immune Checkpoint Therapy in Bladder Cancer. Surg Pathol Clin. 2022; 15:681–94. 10.1016/j.path.2022.07.004. 36344183

[15] Guo CC , Bondaruk J , Yao H , Wang Z , Zhang L , Lee S , Lee JG , Cogdell D , Zhang M , Yang G , Dadhania V , Choi W , Wei P , et al. Assessment of Luminal and Basal Phenotypes in Bladder Cancer. Sci Rep. 2020; 10:9743. 10.1038/s41598-020-66747-7. 32546765 PMC7298008

[16] Raspollini MR , Comperat EM , Lopez-Beltran A , Montironi R , Cimadamore A , Tsuzuki T , Netto GJ . News in the classification of WHO 2022 bladder tumors. Pathologica. 2022; 115:32–40. 10.32074/1591-951X-838. 36704871 PMC10342216

[17] Mohanty SK , Lobo A , Mishra SK , Cheng L . Precision Medicine in Bladder Cancer: Present Challenges and Future Directions. J Pers Med. 2023; 13:756. 10.3390/jpm13050756. 37240925 PMC10222089

[18] Amin MB . Histological variants of urothelial carcinoma: diagnostic, therapeutic and prognostic implications. Mod Pathol. 2009; 22:S96–118. 10.1038/modpathol.2009.26. 19494856

[19] Gandhi J , Chen JF , Al-Ahmadie H . Urothelial Carcinoma: Divergent Differentiation and Morphologic Subtypes. Surg Pathol Clin. 2022; 15:641–59. 10.1016/j.path.2022.07.003. 36344181 PMC9756812

[20] Eturi A , Bhasin A , Zarrabi KK , Tester WJ . Predictive and Prognostic Biomarkers and Tumor Antigens for Targeted Therapy in Urothelial Carcinoma. Molecules. 2024; 29:1896. 10.3390/molecules29081896. 38675715 PMC11054340

[21] Frontiers | Antibody-drug conjugates and predictive biomarkers in advanced urothelial carcinoma. https://www.frontiersin.org/journals/oncology/articles/10.3389/fonc.2022.1069356/full#B35. 10.3389/fonc.2022.1069356 PMC9846350 36686762

[22] Loriot Y , Matsubara N , Park SH , Huddart RA , Burgess EF , Houede N , Banek S , Guadalupi V , Ku JH , Valderrama BP , Tran B , Triantos S , Kean Y , et al. THOR Cohort 1 Investigators. Erdafitinib or Chemotherapy in Advanced or Metastatic Urothelial Carcinoma. N Engl J Med. 2023; 389:1961–71. 10.1056/NEJMoa2308849. 37870920

[23] Zhou TC , Sankin AI , Porcelli SA , Perlin DS , Schoenberg MP , Zang X . A review of the PD-1/PD-L1 checkpoint in bladder cancer: From mediator of immune escape to target for treatment. Urol Oncol. 2017; 35:14–20. 10.1016/j.urolonc.2016.10.004. 27816403

[24] Hanna KS . Advancements in Therapy for Bladder Cancer: Enfortumab Vedotin. J Adv Pract Oncol. 2020; 11:412–17. 10.6004/jadpro.2020.11.4.8. 33604101 PMC7863123

[25] Vranic S , Gatalica Z . Trop-2 protein as a therapeutic target: A focused review on Trop-2-based antibody-drug conjugates and their predictive biomarkers. Bosn J Basic Med Sci. 2022; 22:14–21. 10.17305/bjbms.2021.6100. 34181512 PMC8860310

[26] Loehrer PJ Sr , Einhorn LH , Elson PJ , Crawford ED , Kuebler P , Tannock I , Raghavan D , Stuart-Harris R , Sarosdy MF , Lowe BA . A randomized comparison of cisplatin alone or in combination with methotrexate, vinblastine, and doxorubicin in patients with metastatic urothelial carcinoma: a cooperative group study. J Clin Oncol. 1992; 10:1066–73. 10.1200/JCO.1992.10.7.1066. 1607913

[27] Sternberg CN , de Mulder PH , Schornagel JH , Théodore C , Fossa SD , van Oosterom AT , Witjes F , Spina M , van Groeningen CJ , de Balincourt C , Collette L , and European Organization for Research and Treatment of Cancer Genitourinary Tract Cancer Cooperative Group. Randomized phase III trial of high-dose-intensity methotrexate, vinblastine, doxorubicin, and cisplatin (MVAC) chemotherapy and recombinant human granulocyte colony-stimulating factor versus classic MVAC in advanced urothelial tract tumors: European Organization for Research and Treatment of Cancer Protocol no. 30924. J Clin Oncol. 2001; 19:2638–46. 10.1200/JCO.2001.19.10.2638. 11352955

[28] Sternberg CN , de Mulder P , Schornagel JH , Theodore C , Fossa SD , van Oosterom AT , Witjes JA , Spina M , van Groeningen CJ , Duclos B , Roberts JT , de Balincourt C , Collette L , and EORTC Genito-Urinary Cancer Group. Seven year update of an EORTC phase III trial of high-dose intensity M-VAC chemotherapy and G-CSF versus classic M-VAC in advanced urothelial tract tumours. Eur J Cancer. 2006; 42:50–54. 10.1016/j.ejca.2005.08.032. 16330205

[29] von der Maase H , Hansen SW , Roberts JT , Dogliotti L , Oliver T , Moore MJ , Bodrogi I , Albers P , Knuth A , Lippert CM , Kerbrat P , Sanchez Rovira P , Wersall P , et al. Gemcitabine and cisplatin versus methotrexate, vinblastine, doxorubicin, and cisplatin in advanced or metastatic bladder cancer: results of a large, randomized, multinational, multicenter, phase III study. J Clin Oncol. 2000; 18:3068–77. 10.1200/JCO.2000.18.17.3068. . Retraction in: Ann Oncol. 2011; 22:2536. DOI: 10.1093/annonc/mdr479 PMID: 16807438 11001674

[30] von der Maase H , Sengelov L , Roberts JT , Ricci S , Dogliotti L , Oliver T , Moore MJ , Zimmermann A , Arning M . Long-term survival results of a randomized trial comparing gemcitabine plus cisplatin, with methotrexate, vinblastine, doxorubicin, plus cisplatin in patients with bladder cancer. J Clin Oncol. 2005; 23:4602–8. 10.1200/JCO.2005.07.757. 16034041

[31] Galsky MD , Hahn NM , Rosenberg J , Sonpavde G , Hutson T , Oh WK , Dreicer R , Vogelzang N , Sternberg CN , Bajorin DF , Bellmunt J . Treatment of patients with metastatic urothelial cancer “unfit” for Cisplatin-based chemotherapy. J Clin Oncol. 2011; 29:2432–38. 10.1200/JCO.2011.34.8433. 21555688

[32] De Santis M , Bellmunt J , Mead G , Kerst JM , Leahy M , Maroto P , Gil T , Marreaud S , Daugaard G , Skoneczna I , Collette S , Lorent J , de Wit R , Sylvester R . Randomized phase II/III trial assessing gemcitabine/carboplatin and methotrexate/carboplatin/vinblastine in patients with advanced urothelial cancer who are unfit for cisplatin-based chemotherapy: EORTC study 30986. J Clin Oncol. 2012; 30:191–99. 10.1200/JCO.2011.37.3571. 22162575 PMC3255563

[33] Cathomas R , Lorch A , Bruins HM , Compérat EM , Cowan NC , Efstathiou JA , Fietkau R , Gakis G , Hernández V , Espinós EL , Neuzillet Y , Ribal MJ , Rouanne M , et al, and EAU Muscle-invasive, Metastatic Bladder Cancer Guidelines Panel. The 2021 Updated European Association of Urology Guidelines on Metastatic Urothelial Carcinoma. Eur Urol. 2022; 81:95–103. 10.1016/j.eururo.2021.09.026. 34742583

[34] Balar AV , Castellano D , O’Donnell PH , Grivas P , Vuky J , Powles T , Plimack ER , Hahn NM , de Wit R , Pang L , Savage MJ , Perini RF , Keefe SM , et al. First-line pembrolizumab in cisplatin-ineligible patients with locally advanced and unresectable or metastatic urothelial cancer (KEYNOTE-052): a multicentre, single-arm, phase 2 study. Lancet Oncol. 2017; 18:1483–92. 10.1016/S1470-2045(17)30616-2. 28967485

[35] van der Heijden MS , Sonpavde G , Powles T , Necchi A , Burotto M , Schenker M , Sade JP , Bamias A , Beuzeboc P , Bedke J , Oldenburg J , Chatta G , Ürün Y , et al, and CheckMate 901 Trial Investigators. Nivolumab plus Gemcitabine-Cisplatin in Advanced Urothelial Carcinoma. N Engl J Med. 2023; 389:1778–89. 10.1056/NEJMoa2309863. 37870949 PMC12314471

[36] Food and Drug Administration. FDA approves pembrolizumab for advanced endometrial carcinoma. FDA. 2022. https://www.fda.gov/drugs/resources-information-approved-drugs/fda-approves-pembrolizumab-advanced-endometrial-carcinoma.

[37] Grivas P , Grande E , Davis ID , Moon HH , Grimm MO , Gupta S , Barthélémy P , Thibault C , Guenther S , Hanson S , Sternberg CN . Avelumab first-line maintenance treatment for advanced urothelial carcinoma: review of evidence to guide clinical practice. ESMO Open. 2023; 8:102050. 10.1016/j.esmoop.2023.102050. 37976999 PMC10685024

[38] Powles T , Park SH , Voog E , Caserta C , Valderrama BP , Gurney H , Kalofonos H , Radulović S , Demey W , Ullén A , Loriot Y , Sridhar SS , Tsuchiya N , et al. Avelumab Maintenance Therapy for Advanced or Metastatic Urothelial Carcinoma. N Engl J Med. 2020; 383:1218–30. 10.1056/NEJMoa2002788. 32945632

[39] Siefker-Radtke AO , Necchi A , Park SH , García-Donas J , Huddart RA , Burgess EF , Fleming MT , Rezazadeh Kalebasty A , Mellado B , Varlamov S , Joshi M , Duran I , Tagawa ST , et al, and BLC2001 Study Group. Efficacy and safety of erdafitinib in patients with locally advanced or metastatic urothelial carcinoma: long-term follow-up of a phase 2 study. Lancet Oncol. 2022; 23:248–58. 10.1016/S1470-2045(21)00660-4. 35030333

[40] Loriot Y , Necchi A , Park SH , Garcia-Donas J , Huddart R , Burgess E , Fleming M , Rezazadeh A , Mellado B , Varlamov S , Joshi M , Duran I , Tagawa ST , et al, and BLC2001 Study Group. Erdafitinib in Locally Advanced or Metastatic Urothelial Carcinoma. N Engl J Med. 2019; 381:338–48. 10.1056/NEJMoa1817323. 31340094

[41] Alt M , Stecca C , Tobin S , Jiang DM , Sridhar SS . Enfortumab Vedotin in urothelial cancer. Ther Adv Urol. 2020; 12:1756287220980192. 10.1177/1756287220980192. 33447264 PMC7780177

[42] Guercio BJ , Sarfaty M , Teo MY , Ratna N , Duzgol C , Funt SA , Lee CH , Aggen DH , Regazzi AM , Chen Z , Lattanzi M , Al-Ahmadie HA , Brannon AR , et al. Clinical and Genomic Landscape of FGFR3-Altered Urothelial Carcinoma and Treatment Outcomes with Erdafitinib: A Real-World Experience. Clin Cancer Res. 2023; 29:4586–95. 10.1158/1078-0432.CCR-23-1283. 37682528 PMC11233068

[43] Rosenberg JE , Powles T , Sonpavde GP , Loriot Y , Duran I , Lee JL , Matsubara N , Vulsteke C , Castellano D , Mamtani R , Wu C , Matsangou M , Campbell M , Petrylak DP . Long-term outcomes in EV-301: 24-month findings from the phase 3 trial of enfortumab vedotin versus chemotherapy in patients with previously treated advanced urothelial carcinoma. JCO. 2022; 40:4516. 10.1016/j.annonc.2023.08.016 37678672

[44] Powles T , Rosenberg JE , Sonpavde G , Loriot Y , Duran I , Lee JL , Matsubara N , Vulsteke C , Wu C , Campbell MS , Matsangou M , Petrylak DP . Primary results of EV-301: A phase III trial of enfortumab vedotin versus chemotherapy in patients with previously treated locally advanced or metastatic urothelial carcinoma. JCO. 2021; 39:393. 10.1200/JCO.2021.39.6_suppl.393. 37678672

[45] Tagawa ST , Balar AV , Petrylak DP , Kalebasty AR , Loriot Y , Fléchon A , Jain RK , Agarwal N , Bupathi M , Barthelemy P , Beuzeboc P , Palmbos P , Kyriakopoulos CE , et al. TROPHY-U-01: A Phase II Open-Label Study of Sacituzumab Govitecan in Patients With Metastatic Urothelial Carcinoma Progressing After Platinum-Based Chemotherapy and Checkpoint Inhibitors. J Clin Oncol. 2021; 39:2474–85. 10.1200/JCO.20.03489. 33929895 PMC8315301

[46] Bellmunt J , Necchi A , De Wit R , Lee JL , Fong L , Vogelzang NJ , Climent Durán MA , Petrylak DP , Choueiri TK , Gerritsen WR , Gurney H , Quinn DI , Culine S , et al. Pembrolizumab (pembro) versus investigator’s choice of paclitaxel, docetaxel, or vinflunine in recurrent, advanced urothelial cancer (UC): 5-year follow-up from the phase 3 KEYNOTE-045 trial. JCO. 2021; 39:4532. 10.1200/JCO.2021.39.15_suppl.453.

[47] Necchi A , Fradet Y , Bellmunt J , de Wit R , Lee JL , Fong L , Vozelgang NJ , Climent MA , Petrylak DP , Choueiri TK , Gerritsen WR , Gurney H , Quinn DI , et al. Three-year follow-up from the phase III KEYNOTE-045 trial: Pembrolizumab (Pembro) versus investigator’s choice (paclitaxel, docetaxel, or vinflunine) in recurrent, advanced urothelial cancer (UC). Ann Oncol. 2019; 30:v366–67. 10.1093/annonc/mdz249.018. PMC6594457 31050707

[48] Food and Drug Administration. FDA approves enfortumab vedotin-ejfv with pembrolizumab for locally advanced or metastatic urothelial cancer. FDA. 2023. https://www.fda.gov/drugs/resources-information-approved-drugs/fda-approves-enfortumab-vedotin-ejfv-pembrolizumab-locally-advanced-or-metastatic-urothelial-cancer.

[49] Khaliq N , Sohail M , Younas S , Ishaq M , Akilimali A . Milestone achieved: FDA approves nivolumab in combination with cisplatin and gemcitabine for metastatic urothelial carcinoma. Ann Med Surg (Lond). 2024; 86:6389–92. 10.1097/MS9.0000000000002601. 39525780 PMC11543197

[50] Kim H , Jeong BC , Hong J , Kwon GY , Kim CK , Park W , Pyo H , Song W , Sung HH , Hong JY , Park SH . Neoadjuvant Nivolumab Plus Gemcitabine/Cisplatin Chemotherapy in Muscle-Invasive Urothelial Carcinoma of the Bladder. Cancer Res Treat. 2023; 55:636–42. 10.4143/crt.2022.343. 36228654 PMC10101782

[51] Chu CE , Sjöström M , Egusa EA , Gibb EA , Badura ML , Zhu J , Koshkin VS , Stohr BA , Meng MV , Pruthi RS , Friedlander TW , Lotan Y , Black PC , et al. Heterogeneity in *NECTIN4* Expression Across Molecular Subtypes of Urothelial Cancer Mediates Sensitivity to Enfortumab Vedotin. Clin Cancer Res. 2021; 27:5123–30. 10.1158/1078-0432.CCR-20-4175. 34108177 PMC8634828

[52] Klümper N , Ralser DJ , Ellinger J , Roghmann F , Albrecht J , Below E , Alajati A , Sikic D , Breyer J , Bolenz C , Zengerling F , Erben P , Schwamborn K , et al. Membranous NECTIN-4 Expression Frequently Decreases during Metastatic Spread of Urothelial Carcinoma and Is Associated with Enfortumab Vedotin Resistance. Clin Cancer Res. 2023; 29:1496–505. 10.1158/1078-0432.CCR-22-1764. 36534531 PMC10102834

[53] Rosenberg JE , Powles T , Sonpavde GP , Loriot Y , Duran I , Lee JL , Matsubara N , Vulsteke C , Castellano D , Mamtani R , Wu C , Matsangou M , Campbell M , Petrylak DP . EV-301 long-term outcomes: 24-month findings from the phase III trial of enfortumab vedotin versus chemotherapy in patients with previously treated advanced urothelial carcinoma. Ann Oncol. 2023; 34:1047–54. 10.1016/j.annonc.2023.08.016. 37678672

[54] Bellmunt J , de Wit R , Fradet Y , Climent MA , Petrylak DP , Lee JL , Fong L , Necchi A , Sternberg CN , O’Donnell PH , Powles T , Plimack ER , Bajorin DF , et al. Putative Biomarkers of Clinical Benefit With Pembrolizumab in Advanced Urothelial Cancer: Results from the KEYNOTE-045 and KEYNOTE-052 Landmark Trials. Clin Cancer Res. 2022; 28:2050–60. 10.1158/1078-0432.CCR-21-3089. 35247908

[55] Vuky J , Balar AV , Castellano D , O’Donnell PH , Grivas P , Bellmunt J , Powles T , Bajorin D , Hahn NM , Savage MJ , Fang X , Godwin JL , Frenkl TL , et al. Long-Term Outcomes in KEYNOTE-052: Phase II Study Investigating First-Line Pembrolizumab in Cisplatin-Ineligible Patients With Locally Advanced or Metastatic Urothelial Cancer. J Clin Oncol. 2020; 38:2658–66. 10.1200/JCO.19.01213. 32552471

[56] Powles T , Valderrama BP , Gupta S , Bedke J , Kikuchi E , Hoffman-Censits J , Iyer G , Vulsteke C , Park SH , Shin SJ , Castellano D , Fornarini G , Li JR , et al, and EV-302 Trial Investigators. Enfortumab Vedotin and Pembrolizumab in Untreated Advanced Urothelial Cancer. N Engl J Med. 2024; 390:875–88. 10.1056/NEJMoa2312117. 38446675

[57] Enfortumab Vedotin and Pembrolizumab — A New Perspective on Urothelial Cancer | New England Journal of Medicine. https://www.nejm.org/doi/10.1056/NEJMe2400311. 10.1056/NEJMe2400311 38446680

[58] Powles T , Bellmunt J , Comperat E , De Santis M , Huddart R , Loriot Y , Necchi A , Valderrama BP , Ravaud A , Shariat SF , Szabados B , van der Heijden MS , Gillessen S , and ESMO Guidelines Committee. ESMO Clinical Practice Guideline interim update on first-line therapy in advanced urothelial carcinoma. Ann Oncol. 2024; 35:485–90. 10.1016/j.annonc.2024.03.001. 38490358

[59] Oncology Nursing News. Newer Urothelial Cancer Drugs Are Effective But Pricey. 2023. https://www.oncnursingnews.com/view/newer-urothelial-cancer-drugs-are-effective-but-pricey.

[60] Yu EY , Petrylak DP , O’Donnell PH , Lee JL , van der Heijden MS , Loriot Y , Stein MN , Necchi A , Kojima T , Harrison MR , Hoon Park S , Quinn DI , Heath EI , et al. Enfortumab vedotin after PD-1 or PD-L1 inhibitors in cisplatin-ineligible patients with advanced urothelial carcinoma (EV-201): a multicentre, single-arm, phase 2 trial. Lancet Oncol. 2021; 22:872–82. 10.1016/S1470-2045(21)00094-2. 33991512

[61] Blood-based liquid biopsy: insights into early detection, prediction, and treatment monitoring of bladder cancer | Cellular & Molecular Biology Letters | Full Text. https://cmbl.biomedcentral.com/articles/10.1186/s11658-023-00442-z. 10.1186/s11658-023-00442-z PMC10074703 37016296

[62] Utilization of Circulating Tumor DNA as a Biomarker of Response in a Case of Metastatic Urothelial Carcinoma Treated with Efortumab Vedotin and Pembrolizumab | Published in International Journal of Cancer Care and Delivery. https://journal.binayfoundation.org/article/90317-utilization-of-circulating-tumor-dna-as-a-biomarker-of-response-in-a-case-of-metastatic-urothelial-carcinoma-treated-with-efortumab-vedotin-and-pembro.

[63] Sharp M , Dohme LLC . A Phase 3, Randomized, Open-label Study to Evaluate Perioperative Enfortumab Vedotin Plus Pembrolizumab (MK-3475) Versus Neoadjuvant Gemcitabine and Cisplatin in Cisplatin-eligible Participants With Muscle-invasive Bladder Cancer (KEYNOTE-B15/EV-304). clinicaltrials.gov. 2024. Report No.: NCT04700124. https://clinicaltrials.gov/study/NCT04700124.

[64] Sharp M , Dohme LLC . A Randomized Phase 3 Study Evaluating Cystectomy With Perioperative Pembrolizumab and Cystectomy With Perioperative Enfortumab Vedotin and Pembrolizumab Versus Cystectomy Alone in Participants Who Are Cisplatin-Ineligible or Decline Cisplatin With Muscle-Invasive Bladder Cancer (MK-3475-905/KEYNOTE-905/EV-303). clinicaltrials.gov. 2025. Report No.: NCT03924895. https://clinicaltrials.gov/study/NCT03924895.

[65] Powles T , Park SH , Caserta C , Valderrama BP , Gurney H , Ullén A , Loriot Y , Sridhar SS , Sternberg CN , Bellmunt J , Aragon-Ching JB , Wang J , Huang B , et al. Avelumab First-Line Maintenance for Advanced Urothelial Carcinoma: Results From the JAVELIN Bladder 100 Trial After ≥2 Years of Follow-Up. J Clin Oncol. 2023; 41:3486–92. 10.1200/jco.22.01792. 37071838 PMC10306435

